# Wearables in Medicine

**DOI:** 10.1002/adma.201706910

**Published:** 2018-06-11

**Authors:** Ali K. Yetisen, Juan Leonardo Martinez‐Hurtado, Barış Ünal, Ali Khademhosseini, Haider Butt

**Affiliations:** ^1^ Institute for Measurement Systems and Sensor Technology Technische Universität München Theresienstrasse 90 Munich 80333 Germany; ^2^ School of Chemical Engineering The University of Birmingham Edgbaston Birmingham B15 2TT UK; ^3^ Institute of Translational Medicine Mindelsohn Way, Edgbaston Birmingham B15 2TH UK; ^4^ TUM Incubator Technische Universität München Lichtenberg Str. 6 Garching b. München D‐85748 Germany; ^5^ Triton Systems Inc. 200 Turnpike Rd. Chelmsford MA 01824 USA; ^6^ Department of Bioengineering Department of Radiology Department of Chemical and Biomolecular Engineering University of California Los Angeles CA 90095 USA; ^7^ Nanotechnology Laboratory School of Engineering University of Birmingham Birmingham B15 2TT UK

**Keywords:** biosensors, diagnostics, drug delivery, personalized medicine, telemedicine

## Abstract

Wearables as medical technologies are becoming an integral part of personal analytics, measuring physical status, recording physiological parameters, or informing schedule for medication. These continuously evolving technology platforms do not only promise to help people pursue a healthier life style, but also provide continuous medical data for actively tracking metabolic status, diagnosis, and treatment. Advances in the miniaturization of flexible electronics, electrochemical biosensors, microfluidics, and artificial intelligence algorithms have led to wearable devices that can generate real‐time medical data within the Internet of things. These flexible devices can be configured to make conformal contact with epidermal, ocular, intracochlear, and dental interfaces to collect biochemical or electrophysiological signals. This article discusses consumer trends in wearable electronics, commercial and emerging devices, and fabrication methods. It also reviews real‐time monitoring of vital signs using biosensors, stimuli‐responsive materials for drug delivery, and closed‐loop theranostic systems. It covers future challenges in augmented, virtual, and mixed reality, communication modes, energy management, displays, conformity, and data safety. The development of patient‐oriented wearable technologies and their incorporation in randomized clinical trials will facilitate the design of safe and effective approaches.

## 1 The Rise of Personalized Medicine

**Figure 1:**
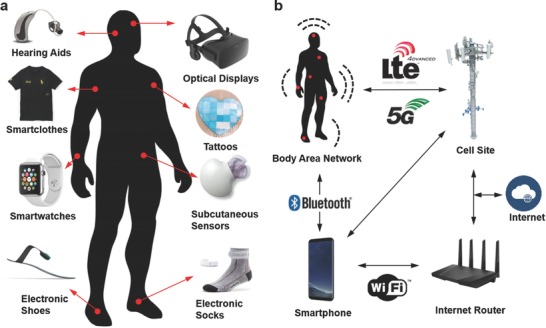
Wearables devices for medical applications. a) Wearable devices (in vitro) have loose or conformal contact with the skin, or worn/inserted through body orifices. The most common interface is loose skin contact wearables, which measure electrophysiological signal via optics and electrodes. b) Information transfer from wearables. The data collected from wearable devices can be transmitted to the Internet or other devices via a body area network, Bluetooth, Wi‐Fi, LTE, 3G, 4G, or 5G connection. The medical data can be sent to a healthcare provider to receive therapeutic feedback or acted upon automatically by other devices in the network.

The “quantified self” movement is the driving force behind wearable technologies involving acquisition of data on daily activities, sport performance, and health status.^1^ In combination with value‐based healthcare systems through telehealth, wearable devices can enable monitoring at risk patients, intervening diseases at an earlier stage, and reducing healthcare expenditures by means of prediction and prevention of disease.^2^ These wearable technologies include smartwatches, wristbands, hearing aids, electronic/optical tattoos, head‐mounted displays, subcutaneous sensors, electronic footwear, and electronic textiles (**Figure** **1**a). They can be conformably placed on the epidermis, inserted through the skin or body orifices for measuring electrophysiological or biochemical signals, and delivering drugs.^3^ Such technologies when incorporated in garments, accessories, or epidermal surface to provide electronic alerts, sense physical and biochemical information, or deliver drugs are broadly called medical wearables.^4^, ^5^

Wearable devices have the potential to offer features such as augmented, virtual, and mixed reality, artificial intelligence, and pattern recognition.^6^, ^7^, ^8^ These ubiquitous computing technologies typically contain microprocessors, sensors, and smartphone interfaces including wireless data communications to record medical data in real‐time and exchange information with other devices and/or centralized databases.^9^ Sensors integrated in wearable devices include inertial measurement units (gyroscopes, accelerometers, barometers, magnetometers), optical sensors (complementary metal–oxide–semiconductor (CMOS) sensors, spectrophotometers, cameras, photoplethysmogram), chemical probes, electrodes, temperature sensors, microphones, shock detectors, strain gauges, and pressure sensors.^10^ Multiple arrays of sensors enable obtaining data from multiple wearable devices and routing to a body area network.^11^ This body area network can transmit the medical data to the Internet through Bluetooth, Wi‐Fi LTE, 3G, 4G, or 5G connection for further analyses or feedback from a healthcare provider (Figure 1b). The capability of wearable devices, smartphones, and wireless communication devices to interoperate in a network infrastructure of connected devices constitutes Internet of things (IoT).^12^ Within this scheme, the medical data can be acted upon within the network or sent to emergency services, medical databases or physician's office to obtain therapeutic feedback about the health status.^13^ The diagnostic information collected from a sensor or an array of sensors can be connected, for example, to a drug delivery system to administer a precise dosage of a medication, or an assistive technology for impaired mobility.

### 1.1 Sensory Information in Wearables

Wearable devices integrated with electronic and optical biosensors can provide real‐time data about the electrophysiological or biochemical status of a patient at point‐of‐care settings or in the clinic. Such biosensors can be embedded in electronic tattoos/stamps, patches, prosthetics, textiles, wristbands, and contact lenses to form conformal contact with biological tissue or bodily fluids. These biosensors may be wirelessly powered or run via lightweight batteries that can be seamlessly integrated in wearable devices. Physical and biochemical data can be wirelessly transmitted to the patient or another wearable device for achieving closed‐loop therapeutic systems.

#### 1.1.1 Close‐Contact Wearables

Conformable mounting of a wearable device on the human skin requires compatibility between the mechanical properties of the compliant wearable device substrate and the stiff surface. Understanding the mechanophysiology at the interface of the wearable device and epidermis is critical in wearable device design, which should adhere to strict design guidelines for the selection of materials and the geometry of the patterns to prevent device failure. The surface of the skin consists of features having 10–1000 µm height and it can be assumed to be a bilayer structure: the epidermis (thickness = 0.05–1.50 mm, Young's modulus = 140–600 kPa) and the dermis (thickness = 0.3–3.0 mm, Young's modulus = 2–80 kPa).^14^ This skin bilayer is linearly elastic for tensile strain values < 15% and enters plastic deformation above 30%. Hence, the mechanical properties of the device materials such as Young's modulus and bending stiffness must be compatible with the mechanical properties of the skin. Elastomers having low Young's modulus (50–100 kPa) are preferred in the fabrication of wearable devices. If the device contains standard electronic components having silicon (160 GPa) or gallium arsenide (90 GPa), the effective moduli can be within the range of 100–150 kPa.^15^ Wearable devices may have a bending stiffness within the range of 1–10 nN m. Low effective moduli and thin devices are ideal to prevent interfacial cracks and interface delamination from the skin. The effective modulus of a close‐contact wearable device can be calculated by approximating the substrate, sensing units, and interconnects. The effective modulus can be approximated as *E*_eff_ = *E*_connect_ (1 + *L*_d_ *L*_s_^−1^), where *E*_connect_ is the effective modulus of the interconnects, *L*_d_ is the characteristic wearable device size, and *L*_s_ is the distance between sensing units or components.^15^ These devices maybe mounted to the skin using a polyvinyl alcohol film support (thickness = 50 µm, Young's modulus ≈1.9 GPa).

**Figure 2:**
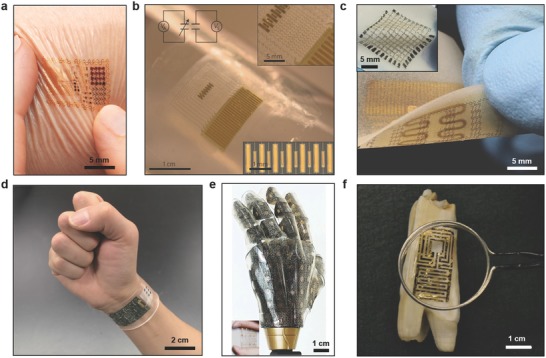
Applications of wearable devices. a) Electronic tattoos conformally attached to the skin via van der Waals forces. Reproduced with permission.^15^ Copyright 2011, American Association for the Advancement of Science. b) A compliant modulus sensor comprising nanoribbons of lead zirconate titanate in arrays of mechanical actuators and sensors. The upper and lower insets show the interconnected array and actuator/sensing regions. Reproduced with permission.^16^ Copyright 2015, Nature Publishing Group. c) A wearable device that can monitor muscle activity, store data, and wirelessly communicate data closed‐loop therapy. The inset shows a wearable RAM array (10 × 10) on the patch. Reproduced with permission.^19^ Copyright 2014, Nature Publishing Group. d) A wearable wristband consisting of electrochemical sensors for the quantification of concentrations of glucose, lactate, electrolytes (Na^+^, K^+^ ions), and temperature for application in real‐time perspiration analysis. Reproduced with permission.^28^ Copyright 2016, Nature Publishing Group. e) Prosthetic skin featuring pressure, strain, humidity and temperature sensors, as well as electroresistive heaters for creating a skin‐like perception. The inset shows the prosthetic skin under ≈20% strain. Reproduced with permission.^32^ Copyright 2014, Nature Publishing Group. f) A antimicrobial peptide‐functionalized graphene sensor on a tooth for wireless detection of bacteria. Reproduced with permission.^47^ Copyright 2012, Nature Publishing Group.

Tattoo‐like films and patches placed over the skin have been utilized as temperature, electrophysiological, and strain sensors (**Figure** **2**a).^15^ Electrical components were constructed on a ≈30 µm gas permeable silicone sheet having low Young's modulus (≈60 kPa) similar to that of the human skin. The elements of the electronic tattoo consisted of conventional semiconductor materials including silicon, gallium arsenide, which were patterned as serpentine ribbons in microscale geometries. The wearable device was elastic under strain deformations having effective moduli of less than 150 kPa and bending stiffness of 1 nN m, thus allowing for it to be mounted on the skin via a temporary support polyvinyl alcohol film. Surface tension forces facilitated the adhesion of the wearable device to the skin having 20% areal contact of electronic components with the skin to establish an electrical interface. These tattoos could incorporate different components including capacitors, light‐emitting diodes (LEDs), transistors, radio frequency inductors, photodetectors, rectifying diodes, and oscillators. Moreover, electronic tattoo electrocardiograms (ECG) distinguished phases of heartbeats such as depolarization of the cardiac wave, and associated QRS waves in an ECG. Other uses of electronic tattoos are noninvasive electromyogram recording on the throat for recognized vocalization of different words.^15^

Wearable piezoelectric tattoos have been fabricated to measure soft tissue viscoelasticity over epidermis (Figure 2b).^16^ A device was constructed from stretchable networks of mechanical actuators, and nanoribbon sensors containing lead zirconate titanate. The device incorporated capacitor‐type components including various piezoelectric layers, which were fabricated on elastomers (20 µm) to achieve stretchable mechanics. Lamination of the wearable piezoelectric actuator–sensor devices allowed for forming conformal contact with soft biological tissues via van der Waals forces, where the bending stiffness per sensor width was 4.5 × 10^−8^ N m. Cycles of stretching over 1000 times showed no delamination of the sensors from the substrate. Measurements of the localized skin properties over lesion sites showed higher modulus (≈6 mPa) than normal skin regions (≈5 mPa) in patients. The wearable device shaped as a protractor allowed quantitatively measuring viscoelastic moduli with spatial mapping for potential applications in the dermatological examination of basal ganglia carcinoma, fibroepithelial polyp, and histiocytoma.^16^ In another study, conformal epidermal sensors were designed for strain detection.^17^ The sensor consisted of LC resonators with capacitive electrodes that responded to variation in skin mechanics. Changes in the LC resonance frequencies were detected by monitoring the absorption of the coil electromagnetic energy, which was linked to an impedance detection device. The device had a resolution of 1.3% in strain detection. This sensor may allow monitoring the mechanical properties of the skin (e.g., lymphedema).^17^

Commercial integrated circuits and hardware can be incorporated in flexible polymer layers. Interconnected assemblies of chips have been created by microfluidic suspension in silicone elastomeric enclosures.^18^ In this assembly, each component was attached to the bottom surface of the enclosure through localized support posts. The electronic components were suspended in a surrounding dielectric fluid to isolate them mechanically. These components were joint to each other via serpentine‐shaped interconnects. The constructed system had the capability of acquisition, filtering, low‐noise amplification, and frequency‐modulated RF transmission of electrophysiological data. The resulting assembly could be stretched (≈30% uniaxial strain) and twisted (≈75%). The final device was laminated over the skin to create a sensory interface. Lamination of the device over the sternum enabled wireless collection of ECG (≈2.4 GHz) at distances up to 1 m. Q, R, and S waveforms were identified with low signal‐to‐noise values as compared to the commercial hardware.^18^

A wearable drug‐delivery device featuring silicon nanomembrane strain sensors, a temperature sensor, a resistive random access memory (RAM) array, and electroresistive heaters has been developed (Figure 2c).^19^ The RAM was formed by depositing TiO_2_ membranes within interstitial gold nanoparticle layers assembled via Langmuir–Blodgett process. A key concept of this wearable technology was energy‐efficient RAM due to the incorporation of gold nanoparticles to extend usage time. The constructed RAM operated at 100 µA, where the data was readable over 100 operations. The RAM did not have significant signal drift post 1000 stretching cycles at ≈30% strain. The sensors were used to monitor the tension and compression cycles on a wrist for potential application in detecting tremor frequencies in Parkinson's disease and epilepsy.^19^ The device was able to log data in different memory cells in every 10 s and the data was read in every 0.5 s. The capability to store data was used to trigger and control drug release. The drug delivery unit was created by forming m‐silica nanoparticles loaded with therapeutic compounds on a polymeric hydrocolloid membrane. An electroresistive heating unit was utilized to transdermally deliver drugs as well as serving as a temperature sensor. As the heat increased in the wearable device, the physical bonding between the nanoparticles and the drug degraded to diffuse the drug transdermally.^19^ This demonstrated wearable device was not wireless; hence, the integration of built‐in microprocessors, wireless communication, and energy transfer/storage units will lead to practical feedback‐based wearable devices.

Tattoo‐based wearables integrating optical sensors have been utilized as indicators for UV radiation exposure. Solar radiation is a risk factor for the major skin cancer forms including cutaneous melanoma and carcinomas (basal and squamous cells).^20^ Hence, the development of wearable sensors is highly desirable for determining the amount of UV exposure to develop awareness about the application of sun screens. La Roche‐Posay (L'Oreal) has recently commercialized a water‐resistant stretchable skin sensor to monitor UV exposure to the skin.^21^ Under the UV exposure, the photosensitive dyes within the 100 µm thick patch change color, which can be read with a smartphone camera.^22^ The patch is air‐permeable and can be worn up to 3 d.

A wearable tattoo‐based sensor was developed to monitor alcohol in sweat.^23^ The device had an iontophoretic‐biosensor along with a flexible electronics unit. Wearables could be a convenient technology to monitor alcohol intake to prevent unsafe drinking. Alcohol consumption results in 3.3 million deaths globally,^24^ and in the United States, ≈25 000 people die each year due to drunk driving.^25^ This device uses sweat secreted by the transdermal delivery of the pilocarpine via iontophoresis. Ethanol was amperometrically measured using a Prussian Blue electrode transducer comprising alcohol oxidase enzyme. The sensor detected blood alcohol concentration within the range of 0.001–0.062%. However, variation in skin permeability and sweat composition limits the practical use of this wearable device. Moreover, a startup company (BACtrack) has designed a wearable sensor that measures the blood alcohol content (BAC) from sweat.^26^ A lag time of ≈45 min was recorded for the alcohol to be present in sweat; hence, this wearable device measures a person's recent history of drinking habits. Another wearable device that can track alcohol is PROOF, which utilizes an enzymatic electrochemical sensor to measure perspired alcohol concentration.^27^ The cartridge of the sensor can be used for continuous measurements of alcohol over 12 h.

Noninvasively monitoring a patient's health status at the molecular level is the key in advancing the applications of wearables. A wearable device that can be worn on wrists or arms has been developed for application in wireless perspiration analysis (Figure 2d).^28^ The device comprised of multiplexed electrochemical sensors on a flexible circuit board for quantitatively monitoring electrolytes and metabolites in sweat. To realize the device, signal transduction, signal amplification, calibration, and wireless data transfer have been integrated within one chip. Glucose and lactate oxidase were immobilized within a chitosan substrate and Ag/AgCl conductor was used as reference counter electrodes. Amperometric detection was used as sensing units within a chitosan film. Prussian blue was used as a mediator to decrease the reduction potentials and activated the sensors without an external power source. Electrolytes were measured with ion‐selective electrochemical electrodes, where poly(3,4‐ethylenedioxythiophene)–poly(styrenesulfonate) performed as an ion‐to‐electron transducer, and CNTs were incorporated within the polyvinyl butyral reference membrane for continuous measurements.^29^ Furthermore, a microcontroller embedded in the wearable device calibrated and compensated the signal drift of the sensors. The data was communicated to a handheld device via on‐board wireless transceiver and Bluetooth connection. The concentrations of monovalent metal ions (Na^+^ and K^+^) as well as glucose and lactate were continuously monitored in a human subject while exercising with a constant load. Although the sweat concentrations of glucose, K^+^ ions and lactate decreased during exercise, sweat Na^+^ ion concentration increased.^28^ These changes might be attributed to the dilution of analyte concentrations. The wearable device was also utilized to determine dehydration status of a group of subjects during a prolonged outdoor running trial. Recently, a wearable patch that integrates biochemical analysis and electrophysiological signals has been fabricated.^30^ By incorporating a three‐electrode amperometric biosensor and a bipolar electrocardiogram sensor on a flexible polyester sheet, real‐time measurements of lactate and heart function was achieved. The wearable patch transferred the monitored signals to a mobile device by Bluetooth low‐energy. Additionally, epidermal patches that can measure pH have been developed for application in the assessment of the wound healing.^31^ Mesoporous microparticles have been functionalized with pH‐sensitive dyes. The electrostatic interactions between the mesoporous structure and the pH‐sensitive dye prevented dye leakage. These particles have been loaded to alginate microfibers via a microfluidic spinning system. The microfibers created conformal contact with the skin and they were able to measure pH within the range of 5.5–7.5.

Challenges exist in the material selection to maintain the conformal contact with the skin. For example, dead cell efflux through the skin may interfere with the measurement data. Another challenge that limits the adoption of electronic tattoos is that long time‐wear over the skin may cause ‘irritant' dermatitis. One potential direction in the development of electronic tattoos is the use of surgical‐grade stainless steel and its alloys that can provide high hypoallergenic features.

#### 1.1.2 Wearables over the Body

The development of electronic prosthetics can allow for sensor‐laden bionic systems having spatiotemporal resolution. An electronic prosthetic skin has been created to incorporate single crystalline silicon nanoribbon pressure, strain, temperature, humidity sensors, and electroresistive heating units for nerve stimulation (Figure 2e).^32^ The sensors were organized in isolated layers and the device mechanically coupled on curvilinear surfaces. This three‐layer artificial skin had localized perception in response to external stimuli to act as a peripheral nervous system interface. The bottom layer of the electronic skin had electroresistive filamentary heating units on a polydimethylsiloxane (PDMS) substrate. The middle layer had temperature, pressure, and strain sensors organized in linear and serpentine patterns. The top layer contained a humidity sensor array comprising coplanar capacitors. Each layer had interconnections to an external microprocessor. The capability of the device to function as a prosthetic skin was tested in different scenarios. For example, the prosthetic skin can map spatiotemporal strain, detect temperature changes (0–65 °C), and measure the humidity level of an object. Additionally, the signals from the prosthetic skin were transmitted to the corresponding peripheral nervous system in a rat model. To create a skin‐nerve interface, multielectrode arrays were coated with platinum nanowires, where ceria nanoparticles were adsorbed to scavenge reactive oxygen species to potentially prevent inflammation. Electrophysiological signals originating from the ventral posterolateral nucleus revealed that the pressure sensor response resulted in synchronized spikes, showing electrical signal transmission to the central nervous system.^32^ A limitation of this system includes the entrance of fractured platinum nanoparticles into the bloodstream and the role of ceria nanoparticles in the inflammation suppression was unclear. Recently, tactile pressure sensors have been developed in the form of active‐matrix arrays consisting of pressure‐sensitive graphene transistors with air–dielectric layers.^33^ These tactile sensors allowed detecting a broad pressure range from 250 Pa to 3 MPa.

#### 1.1.3 Textile‐Based Wearables

Sensors have been incorporated into fabrics to create textile‐based diagnostic devices. The electrodes were sawn into textiles using a computerized embroidery machine in customized geometries.^34^ These embroidered electrochemical sensors have been used for quantitative analysis in biofluids. Glucose and lactate oxidase were immobilized on conductive Ag/AgCl‐coated treads to create these flexible sensors on textiles. As the concentration of glucose was increased from zero to 40 mmol L^−1^ in blood, the current through the embroidered electrodes shifted 0.9 mA.^34^ Additionally, lactate oxidase electrodes that underwent 100 cycles of bending (90°) showed negligible difference in the sensor readouts. In another study, polyurethane‐based ion‐selective membranes were integrated with textile‐based potentiometric sensors to create stretchable diagnostic devices.^35^ The polyurethane membrane and carbon nanotube (CNT) ink was combined with a platinum‐catalyzed silicone film having Ag/AgCl ink and a reference electrode for the quantification Na^+^ and K^+^ ions. A wireless high‐input impedance voltmeter was used to transfer real‐time measurement data to a tablet computer with an iOS application. Na^+^ and K^+^ ion selective electrodes showed a limit of detection of ≈10^−5^ m.^35^ Other sensors integrated within textiles have been utilized for physiological monitoring such as respiration,^36^ heart rate,^37^ and temperature measurements.^38^

#### 1.1.4 Electronic Footwear

The analysis of human walking patterns is required to correct abnormal gaits. An electronic shoe‐based gait monitoring device has been developed to monitor patients with walking problems.^39^ To measure ground contact forces, continuous pressure transducers were utilized with a fuzzy logic algorithm. This algorithm allows the detection of abnormal foot pressure patterns that do not follow natural sequence of gait phases. In another study, electronic shoes have been designed to monitor human motion and body posture.^40^ A mobile 3D motion capture in combination with inertial sensors and electronic shoes was devised to enable diagnosis and analysis of abnormal postures. The measurements of ground reaction forces obtained from the electronic shoes are converted to quaternion in order to prevent gimbal lock. These electronic devices can be configured to harvest energy to power a Bluetooth step counter system.^41^ Such a system may consist of a magnetoinductive transducer for power conditioning to utilize the energy recovery as the shoe impacted on the ground. The energy harvester in the shoe involved exploiting pulse width modulation for performing transducer output ratification and emulating optimal load impedance and charging a storage capacitor. In a typical foot step, the mean energy recovery was 644 µJ. In another study, electronic shoes have been utilized to assess the walking ability of elderly patients with lumbar spinal stenosis.^42^ The electronic shoes had an array of sensors to measure lateral plantar pressure, heel‐strike, and toe pressure with spatiotemporal resolution. These sensors acquired real‐time data at 80 Hz and transmitted this information to a computer via the IEEE 802.15.4 standard. The Oswestry disability index was estimated in a 10 min self‐paced walking test and machine learning algorithms were employed.

Electronic socks have also been developed to monitor vital functions in the body. In one such study, piezoresistive pressure sensors were knitted in a sock to wirelessly measure gate and plantar pressure up to 500 kPa.^43^ The data allowed evaluating running and walking modes of asymptomatic and flat foot. In another study, electronic socks were utilized to prevent pressure‐induced foot ulcers in diabetic patients.^44^ The socks consisted of cotton, polyamide, silver‐coated cotton, and piezoresistive fibers. Silver‐coated fibers knitted to piezoresistive fibers collected and transmitted an electrical signal, which was correlated with pressure values. The real‐time data was stored in a centralized serial memory and sent to a computer wirelessly via Bluetooth. In another study, foot motion was monitored with electronic socks comprising flexible conductive polymers on an elastic textile.^45^ When the socks were subject to strain, the resistance of the conductive polymers were changed. These sensors were utilized to measure foot motion patterns at joint locations. Dynamic input strain was tracked up to 4 Hz. The sensors allowed distinguishing motion around metatarsophalangeal and ankle joints during dorsiflexion. Recently, an electronic shoe consisting of textile sensors connected to a low energy local area Bluetooth beacon has been used to improve athletic performance by optimizing the biomechanics of running.^46^

#### 1.1.5 In Vivo Interface Wearables

Wearable electronic films have been incorporated over the surface of tooth enamel (Figure 2f).^47^ A graphene layer was printed onto water soluble silk allowing for self‐assembly of antimicrobial peptides for bacteria detection in saliva. The device also featured a resonant coil for wireless detection of *H. pylori*, which causes 90% of stomach cancers and duodenal ulcers.^48^ The wireless query operation was achieved by a parallel resonant circuit having a gold‐coated meander line inductor as well as capacitive electrodes. The resistance change was wirelessly monitored as a function of the concentration of bacteria present in saliva. The in vitro studies showed a correlation between bacteria concentration logarithm and variation in resistance showing a detection limit of ≈100 cells.^47^

Ocular diagnostic devices have undergone rapid development over the last decade.^49^ Intraocular pressure can be monitored though measuring the forces acting on the contact lenses to manage glaucoma. To measure intraocular pressure, capacitive,^50^ piezo‐resistive,^51^ mechanical strain gauge,^52^ and microinductor^53^ sensors have been developed. Sensimed AG has introduced a contact lens sensor (Triggerfish) that holds Class IIa CE approval.^54^ The sensor comprised Pt–Ti strain gauges for measuring the dimensional changes over corneoscleral junction for estimating intraocular pressure. An external wearable device attached to the waist of the patient wirelessly powered the Triggerfish contact lens while it received wireless readouts. This contact lens sensor provided readouts up to 288 measurements within 24 h. However, wearing Triggerfish contact lenses produces physiological irregularities as compared to conventional contact lenses, resulting in low oxygen transmissibility to the cornea and altering intraocular pressure measurements.^55^ Another limitation of Triggerfish is that the readouts are in arbitrary units, hence the clinical interpretation of the results are challenging.^56^

The prospect to use contact lenses to quantify tear fluid composition through smartphones or a wearable device has generated interest from Verily, Microsoft, and Novartis.^49^ Blood and tear fluid have a compositional difference due to the blood–tear barrier; however, the concentrations of analytes in both fluids have a relationship as a result of plasma leakage.^57^ Their correlation allows using tears as a surrogate for the continuous measurements of blood chemistry.^58^ Contact lenses have been functionalized with electronics and photonic crystals to report on the concentrations of analytes in tear fluid.^59^ Detection approaches including electrochemical, fluorescence, and photonic crystal probes have been integrated in contact lenses for the continuous monitoring of analytes in tears. Miniaturized electrochemical sensors featuring 3‐electrode systems were coupled with enzymatic reactions for the quantitative measurements in contact lenses.^60^ Such contact lens sensors could be powered by near‐field inductive coupling.^61^ An electrochemical sensor incorporating glucose oxidase enzyme was tested in rabbits.^62^ The contact lens sensor tracked the tear fluid glucose concentration ((0.03–5.00) × 10^−3^ m), which had a 10 min delay time depending on blood glucose. The utilization of gold electrodes featuring 3D pillars increased the surface area (300%) as compared to 2D geometries to achieve a sensitivity of 40 × 10^−6^ m in vivo.^63^ Electronic contact lenses may be configured to provide quantitative or semiquantitative readouts wirelessly or using an LED display. For example, based on the concentration of the glucose, the intensity of an LED embedded in the contact lens can change, where the light output can be quantified using a smartphone camera. Similarly, for semiquantitative applications, for example, when the glucose concentration in tears decreases below 0.9 mmol L^−1^ (threshold value), and the LED in the contact lens can be turned on or off.^64^ Other electrochemical sensors embedded in contact lenses were utilized to quantify lactate concentration in 35 s with 53 µA mm ^−1^ resolution.^65^

Optical sensors including fluorescence probes have been also investigated. Glucose concentration in tear fluid was measured by hydrogel‐coated nanospheres comprising tetramethylrhodamine isothiocyanate concanavalin A and fluorescein isothiocyanate dextran in contact lenses.^66^ These fluorescence probes allowed monitoring tear glucose concentration in a diabetic human patient over 3 h. A photofluorometer via a telemetry transmitter was utilized to send the readouts to an insulin pump.^67^ Additionally, crystalline colloidal arrays and holographic sensors have been incorporated into contact lenses to continuously sense glucose using phenylboronic acid derivatives in hydrogels.^68^ These sensors can be fabricated by exposing a photosensitized hydrogel matrix to laser light interference in Denisyuk holography mode, or self‐assembly of highly charged monodispersed spheres that form close‐packed crystalline arrays in hydrogels via electrostatic repulsion.^69^ Phenylboronic acids form reversible covalent bonds with glucose molecules.^70^ As the concentration of the glucose increases, the phenylboronic acid functionalized hydrogel swells due to Donnan osmotic pressure changes.^71^ Hence, changes in the hydrogel volume results in diffracted Bragg peak shifts, which can be used as an optical transducer to report on glucose concentration in tears.^72^ These reversible optical sensors can quantify a wide range of analytes including pH,^73^ metal ions,^74^ carbohydrates,^75^ and alcohol,^76^ as well as temperature.^77^ Such photonic devices may be also printed in 2.5D format on flexible substrates to sense humidity and glycerol.^78^ Bioinspired optical nanostructures may be also incorporated in contact lenses for application in real‐time sensing.^79^

Microfluidic contact lenses have been fabricated by laser ablation and fiber templating. A microlithography method has been developed to create microconcavities and microchannels in contact lenses.^80^ The microfluidic channel diameters ranged from 100 to 150 µm and their stability properties were evaluated by flow testing of artificial fluid. Microfluidic contact lenses were also functionalized with fluorophores to demonstrate their optical emission capabilities in the visible spectrum. Microfluidic contact lenses may allow sampling tear fluid for multiplexed sensing in vivo and they may simultaneously deliver drugs to the eye.^80^

The capability to monitor glucose concentration in interstitial fluid as a surrogate for blood chemistry allows preventing hypoglycemic episodes and tightly controlling the glucose concentration throughout the day.^81^ Real‐time monitoring of glucose in conjunction with automated insulin pumps lowers the glycated hemoglobin (HbA1c) level, which is an indicator for glycemic control.^82^ Continuous glucose monitoring systems utilize minimally invasive subcutaneous sensors to measure glucose concentration in interstitial fluid via electrochemistry.^83^ Such glucose sensors can communicate with insulin pumps to create a closed‐loop feedback to control the concentration of the glucose in blood (normal: 4.2–6.4 mmol L^−1^, diabetics: >7.0 mmol L^−1^). Commercial continuous glucose monitoring systems include Guardian REAL‐Time (Medtronic), SEVEN Plus/G4 (Dexcom), FreeStyle Navigator (Abbott), HG1‐c (C8 Medisensors), and GlucoTrackT (GlucoTrack). The electrochemical sensor probes (length = 9–14 mm) are subcutaneously inserted into the interstitial fluid at 45° from skin normal. Recently, new interfaces and connectivity options have been introduced to continuous glucose monitoring systems. For example, G5 Mobile CGM System (Dexcom) offers real‐time glucose monitoring that can be monitored through a smartphone, tablet computer, or smartwatch via Bluetooth. The smartphone application sends audio alerts to the patient when the concentration of the glucose in interstitial fluid is low or high. The information can also be displayed on Apple Watch and Fitbit smartwatch. The development of smartwatch applications triggered open‐source projects (e.g., NightScout movement), in which patients develop their own medical applications to access their continuous glucose measurement data using AndroidWear.^84^ The readout data can be monitored by others or shared with healthcare professionals. Apple health kit, for example, was developed in response to these requirements of connecting devices for gathering medical data in a secure environment, and under complete transparency for healthcare professionals and regulatory authorities; and used in wearable glucose monitoring trials.^85^

### 1.2 Limitations in Existing Sensing Technologies

Electrochemical sensors degrade in vivo due to biofouling, which requires replacement every 3–4 d.^86^ These electrochemical sensors, typically used for the management of diabetes, are intrinsically prone to drift in vivo; hence they require frequent calibration with finger prick blood tests. The measurement of glucose concentration in interstitial fluid is associated with a lag time as compared to blood glucose. Limitations of these wearable glucose sensors apply to all types of electrochemical sensors and include (i) frequent calibration of the sensor, (ii) sensor readout drift due to biofouling, (iii) biweekly sensor replacement, and (iv) relatively high cost. Other well‐known sensors in wearables include heart rate monitors, thermometers, and accelerometers. These types of sensors have been fully characterized and work seamlessly; however, the amount of signal processing and data processing required to for healthcare applications is large and requires continuous operation and energy supply.

### 1.3 Next Generation Sensors

Power‐efficient sensors are increasingly gaining significance in wearables. Recently, a low‐power temperature sensor was developed based on complementary temperature dependencies of n‐ and p‐type metal‐oxide‐semiconductor field effect transistors in the subthreshold region.^87^ Tunneling currents and a capacitive charging‐time‐to‐digital feedback allowed digitizing the temperature at 113 pW, operating from 10 to 60 °C.^87^ Such individual sensors can be interconnected within wearable body area networks.^88^

**Figure 3:**
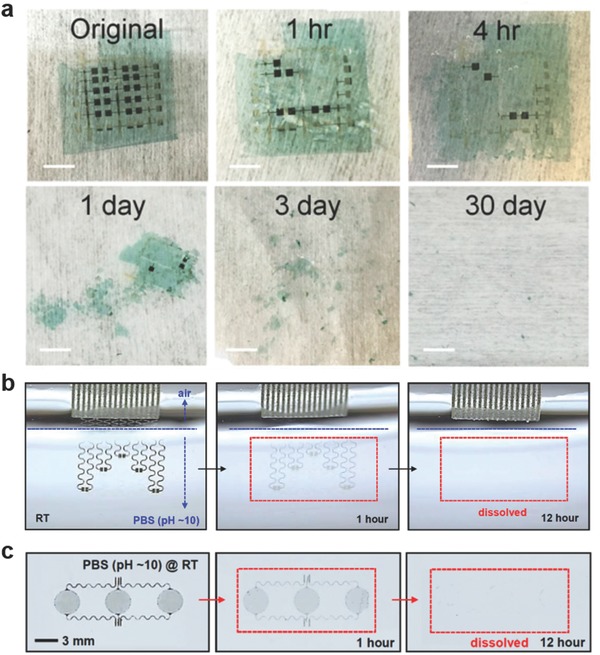
Transient electronics. a) Disintegrable electronics comprising iron as electrodes at various stages of disintegration in a pH 4.6 buffer solution (scale bars = 5 mm). Reproduced with permission.^90^ Copyright 2017, United States National Academy of Sciences. b) A biodegradable pH sensor based on doped silicon nanoribbons (Si NRs) at different stages of dissolution while submerged in PBS (pH 10) at 24 °C. Reproduced with permission.^91^ Copyright 2015, United States National Academy of Sciences. c) Capacitive biodegradable electrophysiological sensors and their dissolution in PBS (pH 10) at 24 °C. Reproduced with permission.^91^ Copyright 2015, United States National Academy of Sciences.

Transient technology is an emerging field and it refers to a group of devices that dissolve over a period of operation.^89^ This may allow practical disposability properties at point‐of‐care settings. After the exposure of the transient device to a stimulus, the functionality of the device is controllably terminated in a specific period of time. The applications of these transient devices have been realized in bioelectronics, environmental monitoring, and energy harvesting. The transient behavior of such devices can be initiated primarily by aqueous solutions, but also at a lesser extent with light, temperature, or mechanical forces. The main substrate building blocks may consist of water‐soluble polymers such as polyvinylpyrrolidone (PVP), poly(vinyl alcohol), polylactic acid, polylacticcoglycolic acid (PLGA), and polycaprolactone (PCL). In transient electronic devices, magnesium (Mg), zinc (Zn), iron (Fe), tungsten (W), and molybdenum (Mo) have been explored as degradable conductive materials. Recently, a transient material based pseudo‐CMOS was demonstrated.^90^ The 800 nm thin device consisted of a degradable cellulose substrate and iron electrodes with thermal and chemical stability to achieve and operating voltage of 4 V (**Figure** **3**a). In a recent study, transient materials have been developed for pH and electrophysiological sensing (Figure 3b,c).^91^ A capacitive electrophysiological sensor was connected to a preamplifier having low‐input capacitance and high‐input impedance. Near‐unity gain was achieved in the preamplifier enabling electrophysiological measurements via a SiO_2_ insulation layer. The amplifier and filter units of this transient device provided tunable gain from 60 to 80 dB for electrocardiogram and electromyogram measurements, where the results were comparable to conventional gel‐based electrodes.

A biodegradable electronic patch integrated with flexible heaters was developed to deliver drugs on demand.^92^ This thermally‐controllable patch allowed administering antibiotics. A blend of poly(glycerol sebacate)‐PCL was synthesized and electrospun to create elastic sheets (tensile modulus = 4–8 MPa), where the fiber diameters ranged from 350 to 1100 nm. A metallic heater was deposited on the elastic sheet, which released antibiotics upon thermal stimulation. In vitro studies showed that the patch successfully released cefazolin in agar plates inoculated with *Staphylococcus aureus* and *E. coli*. Wound patches (1.5 cm in diameter) having heaters resulted in a zone of inhibition up to 1.0 cm over 24 h as compared to control samples without a heater.^92^

## 2 Utilization of Wearables Sensory Data

### 2.1 Management of Wearables Data

#### 2.1.1 Data Storage and Communication

**Figure 4:**
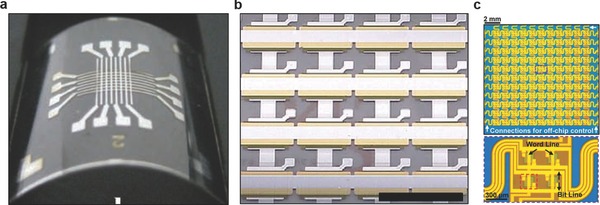
Advances in flexible data storage and memory units. a) Photograph of a flexible 8 × 8 array‐type Ti/Au/Al/PI:PCBM/Al organic memory device. Reproduced with permission.^97^ Copyright 2010, Wiley‐VCH Verlag GmbH & Co. KGaA, Weinheim. b) A flexible 1D‐1R organic resistive memory cell array on a flexible PEN substrate. Scale bar = 5 mm. Reproduced with permission.^98^ Copyright 2013, Nature Publishing Group. c) Photograph of a 22 × 22 multiplexed charge trap floating gate memory array (top) and a magnified image (bottom) illustrating four memory pixels interconnected with word and bit lines. Reproduced with permission.^99^ Copyright 2016, American Association for the Advancement of Science.

Commercial solid‐state, nonvolatile flash memories are based on rigid silicon data storage units. In this scheme, the data is first converted into a charge level and stored in floating gates.^93^ Recent advances in the memory development focused on improving flexibility and stretchability, which are required in medical wearables. Flexible NOR type resistive random‐access memories having one transistor–one memristor (1T–1M) structure have been developed.^94^ These memory cells were created by integrating single crystal silicon transistors with an amorphous α‐TiO_2_ memristor on a flexible substrate. Word, bit, and source lines allowed interconnecting the 1T–1M RRAM unit cells in the 8 × 8 NOR‐type array to achieve independent cell control. Microcontact printing has been utilized to fabricate a flash memory based on a high‐density Ag nanoparticle charge trapping layer on a flexible polyethylene terephthalate (PET) substrate.^95^ A close‐packed Ag nanoparticle monolayer was embedded at the interface of a blocking layer (200 nm) and a 10 nm atomic layer‐deposited Al_2_O_3_ (tunneling layer) to fabricate a data storage unit controlled by external gate bias. As compared to memory devices featuring a floating gate, a memory window of 16.5 V and retention time of 10^5^ s was achieved. The produced devices had high‐density storage sites and low lateral change leakage, as well as offering high endurance over 1000 cycles and robust mechanical stability over 500 bending cycles.^95^ Another study involved the development of flexible transistor organic memory devices consisting of (spin‐coated) crosslinked PVP films and organic pentacene thin‐film transistors (TFTs) on flexible matrices, in which Au NPs were utilized as charge trapping elements.^96^ The produced TFT‐based nonvolatile floating gate device had a memory windows of 10 V and sustained operation over 1000 bending cycles times. Such organic memories can also be fabricated from polyimide (PI) and 6‐phenyl‐C61 butyric acid methyl ester (PCBM) on flexible PET substrates (**Figure** **4**a).^97^ Furthermore, flexible all organic vertically‐stacked 64‐bit memory cell arrays consisting of one diode‐one resistor system without crosstalk have been developed (Figure 4b).^98^ The memories components comprised of poly(3‐hexylthiophene) (P3HT) diodes and a combination of polystyrene (PS) and PCBM on polyethylene naphthalate (PEN) substrates. A photocrosslinker (bis‐perfluorobenzoazide:bis‐FB‐N_3_) was utilized to covalently bind the P3HT and PS side chains by nitrene‐based N‐H insertion. The memory cell arrays were stable (*I*_ON_/*I*_OFF_ ratio ≈10^3^) and had retention time of 10^4^ s, displaying an endurance cycle of 10^2^.^98^ In a recent study, stretchable silicon nanomembranes circuits incorporate silicon nonvolatile memory arrays with nanocrystal floating gates in a wearable device (Figure 4c).^99^ Other emerging data storage technologies include piezoelectricity and inverse magnetostriction.^100^ These nonvolatile memories operate at low energy schemes with storage densities up to 0.2 µm^2^. Recently, ferroelectric based nonvolatile flip flop memories (1.6 µs for 10‐year data retention, and 170 ns for 10 h data retention) were developed to reduce power dissipation for heart rate data storage.^101^

Standard communication protocols for electronic devices include Bluetooth classic,^102^ Zigbee,^103^ and Wi‐Fi.^104^ However, these protocols have not been designed to be power‐efficient. Hence, early wearable device designs included proprietary protocols to reduce energy consumption, but this approach restricted the use of interoperability in a personalized network area or IoT.^105^ To address these issues, Bluetooth low energy (BLE) was developed to achieve power efficiency for short‐range communication while operating within industrial, scientific, and medical (ISM) radio band (2.4 GHz) with a bandwidth of 1 Mbps.^106^ This protocol allows transferring state information in small blocks of data at regular intervals, where the processor operates in a low power mode.^107^ It also supports Adaptive Frequency Hopping with a 32‐bit cyclic redundancy check,^108^ and the beaconing (advertising mode) in the BLE standard allows short, unsolicited messages at flexible update rates.^109^ Thus, it provides a low power high‐rate always connected transfer of data. Always‐connected devices such as smartphones and IoT networks can serve as hosts for BLE‐enabled wearables.^110^ A recent advance in such enabling technologies is Bluetooth 5, which offers quadruple data communication range with increased transmission power, and double the data speed (2 Mbps) as compared to Bluetooth 4.x.^111^

Body area wireless networks are currently being standardized.^112^ For example, the “400 MHz” band comprising Medical Implant Communication Serive band and ISM band are standardized under IEEE 802.15.6 for close‐to‐body wearables, also for low‐power system‐on‐chip architectures.^113^ An emerging problem with sensor networks is the interference with existing Wi‐Fi systems that require more power.^114^ Standards such as IEEE 802.11ah on other bands are adopted for “DeepSleep” power saving mode and energy harvesting.^115^ BLE, ZigBee, the standard 2.4 GHz personal area network radios will still be used for independent sensor connectivity with healthcare platforms and the IoT.^116^ Such interconnected sensors will require safety measures to protect the personal information and patient data.

Wearable devices need to be able to function with an independent operating system offering low power consumption and user friendliness without being paired to smartphones. Android Wear 2.0 (Google) features new functions such as Google Fit and enhanced Google assistant, as well as Android pay via NFC and Smart Reply.^117^ For example, Samsung Gear S offers a micro SIM card option, run applications, and able connect to Wi‐Fi; however, it is only compatible with Samsung smartphones.^118^ In combination with upcoming 5G connectivity, that could enable laptops or IoT devices as connecting hubs, power‐efficient medical wearables could access these connected nodes inside homes, clinics, or transportation systems.^119^

#### 2.1.2 Data Processing and Interpretation

Real‐time data acquisition in wearables requires continuous processor operation, which demands power consumption and thus low power domain sensors. Node processors for sensor signal acquisition offer low power consumption and energy management.^120^ Fine‐grained power management can be achieved via clock gating, which eliminates switches in the processor to improve power efficiency.^121^ Having multiple CPUs for different applications or parts of the feature‐OS together with processors with low leakage can provide power‐efficient wearable devices.^122^ Within such systems, low internal clock speeds and input/output signal processes can be minimized to reduce the overall power consumption. Chip architectures are also being designed to operate at power down modes and adapt high‐speed logic.^123^ For example, Snapdragon Wear 1200 is a low‐power, global navigation satellite system‐enabled processor (Qualcomm) that supports LTE Cat‐M1 (eMTC) and NB‐1 (NB‐IoT) for application in wearables.^124^

#### 2.1.3 Data Safety

The protection of the personal information and patient data in wearables is a significant concern. The ownership of the data from wearable devices is debatable. Currently some wearable device service providers limit their users' access the access to the collected and stored data. These service providers charge their users' fees to access their row data, which is also acquired by third‐party companies. These third party companies also sell patient information (age, sex, height, weight, location, contact details) and global positioning‐tracked activities.^125^ While some manufacturers claim to anonymize patient data by removing identifying features, these protective approaches are inadequate to prevent identity fraud. Advanced algorithms have the capability of crossreference biometric information collected with wearable devices based on users' behavior (activity time, location) to reveal the patient's identity.^126^ For example, digital traces of patient information can be collected by social media to predict identity.^127^ Furthermore, wearable device can be hacked by accessing the communication channel between the wearable device and smartphones.^128^ Such security vulnerabilities have been previously observed in pacemakers and glucose pumps.^129^

Wearable device manufacturers and IT infrastructure should protect medically relevant data by providing tamper protection, authentication, data encryption, or end‐to‐end data integrity. Tamper‐resistant authentication protocols should be lightweight to be integrated into wearables.^130^ Digital cryptography requires computing power and is not available in wearable device applications. Physical cryptography offers an alternative strategy by using physical unclonable functions.^130^ Moreover, authentication protocols can be extended to multiple party interactions, for example, user‐device, device‐network, and user‐network systems.^131^ Body signals may also be used to authenticate and create secure communications.^132^ Additionally, lightweight on‐chip loadable data encryption protocols have been designed for wearable devices.^133^ Preservation of data integrity from end‐to‐end user can be compromised by multiple encryption methodologies that can cause data loss.^134^

### 2.2 Aided Living and Treatment with Wearables

Wearable devices have application in aiding patients with disabilities and metabolic disorders. These applications range from aiding hearing to drug delivery. Wearables can be configured to deliver drugs through skin, eye, or ear interfaces. Their simple formats can actuate the release of therapeutics via mechanical stress or strain. These so called passive wearable devices consist of drug reservoirs directly embedded in the polymer matrixes or microneedle depots. When contacted with the body surface, drugs can be delivered by means of passive diffusion or electronic actuation. Such mechanisms can be triggered by heating, pH, or other chemical means to actuate a drug‐impregnated medium or mechanically opening the depots to initiate drug diffusion. These wearable devices can be integrated with biosensors to create closed‐loop systems, in which the drug dosage can be controlled and wirelessly communicated to the user, and further connected to body area networks for tracking and monitoring.

#### 2.2.1 Aided Living

Hearing impairment is the major reason of disability among old patients.^135^ One of the early examples of the use of wearable devices in medicine was to treat hearing loss. Electroacoustic devices amplify sound and conformably fit behind auricle and/or in the ear canal for the correction of impaired hearing. These devices amplify quiet sounds audible, but a threshold mechanism prevents the amplification of loud sounds. Such devices consist of an earmold, which may contain an electronic circuit, a microphone, a loudspeaker, and a battery (zinc–air, 1.35–1.45 V). However, recent miniaturized models offer devices that can be inserted in the outer ear bowl or the ear canal. Hearing aids can be wirelessly connected to smartphones or tablet computers to directly tune the volume or the bass of the sound.^136^ These wireless hearing aids can communicate with one another to adjust the audio properties simultaneous. Such devices offer FM listening devices with wireless microphones that can be used by a partner. These devices are examples of how wearables can interconnect. Additionally, these devices can connect to the mobile phones, audio sources, and TV streamliners via Bluetooth (2.4 GHz).^137^

#### 2.2.2 Drug Delivery

**Figure 5:**
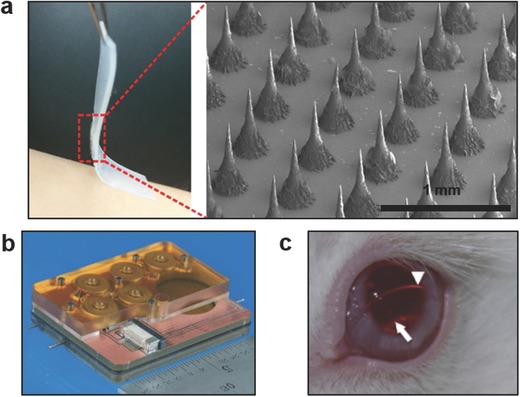
Applications of wearable devices in drug delivery. a) Stretch‐actuated drug delivery from elastomer films comprising microgel depots containing therapeutic nanoparticles for diabetes, anticancer, and antibacterial treatments. The device comprises a microneedle array (SEM image) for stretch‐mediated control of insulin delivery. Reproduced with permission.^138^ Copyright 2015, The American Chemical Society. b) A microfluidic reciprocating pump and electromagnetic actuators for intracochlear drug delivery. Reproduced with permission.^148^ Copyright 2016, The Royal Society of Chemistry. c) Latanoprost‐eluting contact lenses on the surface of a rabbit's eye for glaucoma treatment. The arrowhead illustrates the lens edge and the arrow show inner diameter of drug‐polymer film. Reproduced with permission.^149^ Copyright 2014, Elsevier.

Therapeutic agents can be released from wearable elastomer films by applying mechanical forces. A wearable device containing a microgel depot has been developed to release drug loaded nanoparticles based on tensile strain triggering of an elastomer membrane (**Figure** **5**a).^138^ When tensile strain was applied to the elastomer film, the drug was released due to the Poisson's ratio‐induced compression of the microgel depot and the enlarged diffusion surface area. The wearable device could be configured to release antineoplastic and antibacterial agents based on routine body motions, or pulsatile release by patient‐controlled administration. The effectiveness of the doxorubicin‐eluting film was demonstrated by inhibiting tumor spheroid, showing two times more reduction in tumor size as compared to passive drug release. Furthermore, ciprofloxacin was incorporated into the elastomer film treating local infection on a finger joint. The amount of ciprofloxacin released after 100 and 1000 finger movements was 9.2 and 56.6 µg mL^−1^. Additionally, crosslinked hyaluronic acid microneedles were integrated within the wearable device to transdermally deliver insulin to control the concentration of blood glucose in type 1 diabetic mice. After 10 cycles of stretching at a strain of 50%, the concentration of the blood glucose, which was at a hyperglycemic state (550 mg dL^−1^), decreased to the normal glycemic state (<200 mg dL^−1^) within 30 min.^138^ To refill this drug delivery system, the microgel depot layer may be replaced while retaining the microneedles in situ.

Engineering biocompatible and flexible microneedles will minimize the risks of immune reaction and breakage after skin penetration. Microneedles made of high Young's modulus materials such as silicon, nickel lack biocompatibility. However, microneedles created from polymers have low mechanical strength limiting their application in drug‐eluting patches. The microneedles could be configured to dissolve in vivo. For example, microneedles have been utilized to deliver influenza virus vaccine and dissolve in the skin within minutes.^139^ Recently, a wearable patch comprising a bendable microneedle array was developed.^140^ Each microneedle consisted of four bendable PDMS pillar bases and a rigid SU‐8 or maltose sharp tip. The array tolerated the deformation associated with buckling and lateral movement forces during skin penetration and daily wear without needle breakage. The concentration of blood glucose in rats treated with the insulin‐eluting microneedle array decreased from over 5.5 h, where the dose was controlled from 50 to 183 µL by varying the pressing force.^140^ This drug delivery system was integrated with a refillable microfluidic drug reservoir that was assembled at the backside of the microneedle patch.

A microneedle‐based immunopatch has been created for melanin‐mediated cancer treatment.^141^ Polymeric microneedles consisted of B16F10 whole tumor lysate containing melanin. The microneedles released lysate upon insertion into the skin in a controlled manner. Excitation of the melanin with near‐infrared light increased the local temperature that promoted tumor‐antigen uptake by dendritic cells to improved antitumor vaccination. This patch provided spatiotemporal immunotherapy that enhanced infiltration of polarized T cells and local cytokine release. Studies in mice showed increase in survival after tumor challenge targeting established primary tumors as well as distant tumors.^141^

Transdermal drug delivery has been achieved through iontophoresis in graphene‐based wearable devices.^142^ A thermally controlled transfer printing approach has been developed to produce patterned graphene layers on an elastic stamp. Graphene‐based iontophoresis electrodes was used a heating unit (22–44 °C) to actuate the thermal transdermal drug delivery. Iontophoresis electrodes were laminated on the skin of a nude mouse and doxorubicin was loaded on the graphene electrodes and electric field was applied. The penetration of the depth of doxorubicin was proportional to the applied heat to graphene and the concentration of iontophoresis stimuli.^142^ Such wearable devices may also consist of nanofibers that can be designed in stretchable formats.^143^ These devices many reach sheet resistance of ≈1.3 Ω sq^−1^ having a power efficiency of 0.65 W cm^−2^ and an optical transmittance of ≈90%.

A textile dressing has been developed for temporal and dosage‐controlled drug delivery.^144^ The wound dressing consisted of composite fibers having a core electrical heater, which was coated with an alginate (Alg)/poly(ethylene glycol) diacrylate (PEGDA) film to deliver a drug. The wound dressing allows releasing antibiotics and vascular endothelial growth factors (VEGF) to reduce bacterial infection and induce angiogenesis in vivo. To construct the fibers, a cotton thread was coated with a carbon ink. For example, a conductive tread having a diameter of 1.2 mm showed 5 Ω cm^−1^. The temperature of the fibers could be controlled between 25 and 45 °C as the applied voltage was increased up to 4.5 V. p(NIPAM/PEGDA) hybrid particles containing cefazolin and vancomycin have been synthesized. For example, a 5 cm long thread released 30 µg cefazolin over an hour, whereas four threads released 130 µm cefazolin. In a diabetic mouse model, fabricated fibers were used to deliver VEGF to skin wounds of the animals to evaluate wound healing rate. Threefold increase in the granulation of the tissue deposition across the wound bed was measured as compared to the control group without VEGF.^144^ These textile dressings may be integrated with hydrogel optical fibers and photonic nanomaterials for photodynamic therapy.^145^

Microfluidic systems have been developed to deliver drugs to inner ear fluid.^146^ A microfluidic device has been fabricated to infuse and withdraw sub‐microliter drug solutions to and from inner ear fluid to achieve a liquid transfer with zero net volume.^147^ The drug delivery system consisted of a polymer reciprocating pump and an electromagnetic actuator for application in head‐mounted wearables. The mass transport of the drug molecules to the cochlea was achieved through diffusion and mixing. The programmable pump created a reciprocating flow and a reservoir to control the drug concentration in the infused bolus. The efficacy of the drug delivery device was evaluated by delivering 6,7‐dinitroquinoxaline‐2,3‐dione to the cochlea of guinea pigs. After the implantation of the cannula by the 24 kHz region at the cochlea base, auditory nerve compound action potentials were monitored. As the reciprocating pump drove the drug at 0.64–1.18 µL infuse‐withdraw in 3–4 min intervals, compound action potential thresholds increased for 1 h and returned to normal levels.^147^ A recent iteration of the device included an embedded drug reservoir and all fluidic components in the microfluidic architecture (Figure 5b).^148^ This intracochlear drug delivery system operated at 6 s drug loading, 16 s infusion, and 10 min idle stages, where the energy consumption was 20.9 J. This device allowed for running 157 cycles on a 3.8 V 240 mAh power source corresponding to 27.3 h run time. This miniaturized system may also allow for replacing the drug reservoir cartridge for long‐term use.

A contact lens has been created to deliver latanoprost to the eye for the treatment of glaucoma.^149^ The contact lens formulation consisted of latanoprost‐PLGA films encapsulated by methafilcon, which is a co‐polymer of poly(2‐hydroxyethyl methacrylate) (pHEMA) and methacrylic acid. The contact lens (dry thickness = 300 µm, wet outer diameter = 15 mm, wet central aperture = 4 mm) was fabricated by a combination of spincoating, and UV‐initiated free radical polymerization, and lathing the material into a curved geometry. The amount of the drug (89–178 µg) encapsulated in the contact lenses was controlled by varying the concentration of the latanoprost during UV polymerization. The drug was released by an early burst followed by sustained release over a month. 50–90% of the drug amount was released in vitro over 3 d depending on the thickness of the contact lens. The efficacy of the drug‐eluting contact lens in the treatment of glaucoma was assessed by in vivo studies in rabbit eyes (Figure 5c). The concentration of the latanoprost released from 3‐d preconditioned contact lenses decreased from 100 to 10 ng mL^−1^ over 3 d, followed by a sustained release over a month.^149^ Recently, enzyme‐cleavable contact lenses were developed.^150^ These lenses comprised nanodiamonds that released timolol maleate in the presence of lysozyme for sustained glaucoma treatment. The nanodiamonds were coated with polyethyleneimine and crosslinked with an enzyme‐cleavable chitosan to encapsulate timolol maleate. The nanodiamond drug reservoirs were embedded in a pHEMA matrix and casted to obtain a contact lens. In the presence of lysozyme, the contact lenses released 9.41 µg timolol maleate over a day.^150^ This approach allowed for wet storage of the contact lenses prior to use. Additionally, ciprofloxacin‐eluting contact lenses inhibited *S. aureus* for extended zero‐order release.^151^ These antimicrobial PLGA‐pHEMA contact lenses completely inhibited 10^5^ *S*. *aureus* cells as the cumulative mass release of ciprofloxacin was 4.5 mg over 28 d. In another study, econazole‐eluting PLGA‐pHEMA contact lenses have been fabricated for the treatment of fungal ocular infections.^152^ The antifungal drug‐release kinetics of the contact lenses was tested in an assay against *Candida albicans*. The contact lenses comprising 16 mg econazole killed 100% of the Candida (0.5–1.0 × 10^7^ cells) over 8–10 d.

#### 2.2.3 Behavior Therapy

With the emergence of optical headsets such as Google Glass 2, Oculus Rift and Magic Leap, HoloLens for application in video games and entertainment, reality simulation platforms have also found use in medical research, diagnostics, and treatment.^153^ These simulated reality platforms provide imaginary environment, sounds, vibrations, and other sensations to observe and interact with the imaginary surroundings and items.^7^, ^154^ They can facilitate the management of mental and anxiety disorders including autism, posttraumatic stress disorder (PTSD), persecutory delusions, and phobias.^155^ Enabling patients to evaluate these mental challenges in a virtual reality social environment can allow the management of safety‐seeking or anxiety behaviors. Other applications of reality simulation programs include ocular treatment, rehabilitation, pain management, and surgical training.

Brain power system for autism is a customized head‐mounted display that provides personalized coaching experiences via a gamified augmented‐reality application incorporating artificial intelligence modes.^6^ This application aids the patient to establish emotion recognition, eye contact, face‐directed gaze, and behavioral self‐regulation. The head‐mounted display integrating the brain power system was utilized to coach two children with clinically diagnosed Autism spectrum disorder. 24 h postintervention, these patients demonstrated decreased disorder symptoms based on the aberrant behavior checklist.^156^ In another study, head‐mount displays were utilized to automatically recognize facial expressions.^157^ The headset behaviorally aided children with Autism spectrum disorder by providing real‐time social cues and minimizing distractions. This system displayed facial expressions and records social responses including eye contact time and using an eye tracker. A trial involving 38 children with Autism spectrum disorder and typically developing children showed correct emotion recognition rates within the range of 85–95%. While significant real‐time physiological and behavioral data can be collected from mentally ill patients, the connection between the data and the emotions of the patient is unclear to draw definite diagnosis conclusions. Hence, the interpretation of the results for actionable treatment is a challenge. In another study, a randomized, double‐blind, placebo‐controlled study of virtual reality exposure therapy was designed to treat PTSD due to military trauma in veterans (*n* = 156).^158^ Virtual reality exposure therapy was introduced to patients in six sessions to decrease the PTSD symptoms in combination with psychiatric medications, d‐cycloserine (50 mg) and alprazolam (0.25 mg), and compared to the placebo effect. The virtual reality therapy augmented in reducing PTSD symptoms, and these reduced symptoms were maintained at 3–12 months of treatment.^158^

In a recent study, clinically diagnosed patients with persecutory delusions (*n* = 30) were enrolled to virtual reality cognitive therapy sessions in order to decrease in delusional conviction and real‐world distress.^159^ Virtual reality cognitive therapy reduced delusional conviction by 22% and real‐world stress by 19.6% as compared with virtual reality exposure group.^159^ In another study, VR was also used in treating glossophobia (fear of public speaking) by exposing subjects to a VR public speaking scene over 5 weeks.^160^ Questionnaires such as attitude toward public speaking and subjective units of disturbance, as well as heart rate monitoring showed significant improvements in public speaking fear as compared to a control group.

**Figure 6:**
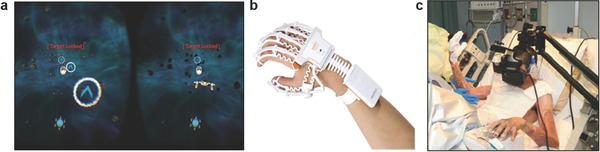
Applications of virtual reality in medicine. a) Dichoptic training game seen through a head‐mounted optical display in virtual reality. The amblyopic eye views the left side of the image to fly the spaceship through the blue gates. Spaceship is only seen with the dominant eye (right side). Reproduced with permission.^161^ Copyright 2017, Springer. b) The RAPAEL Smart Glove system tracks the posture and the motion of a user's distal limb. Reproduced with permission.^138^, ^162^ Copyright 2016, BioMed Central Ltd. c) The use of head‐mounted optical displays with virtual reality in a pediatric burn patient during motion exercises. Reproduced with permission.^163^ Copyright 2014, Mary Ann Liebert, Inc.

Virtual reality head‐mounted displays were utilized to treat patients with amblyopia.^161^ Human subjects (*n* = 17) with clinically‐diagnosed anisometropic amblyopia were enrolled in a 8‐session dichoptic game‐based training program (diplopia, vivid vision), which was operated on a Oculus Rift OC DK2 display. The game involved flying a spaceship through a rings system, where the simulated system was in a 3D dichoptic virtual reality setup (**Figure** **6**a). The virtual vehicle was only observed with the dominant eye and all the objects in the game were seen with the amblyopic eye to force the brain to cooperatively use both eyes. After the treatment, the corrected visual acuity of the patients significantly improved from 0.58 ± 0.35 (LogMAR) to 0.43 ± 0.38 (*p* < 0.01), while mean stereoacuity shifted from 263.3 to 176.7 of arc, showing potential for the treatment of sight disorders.^161^

The rehabilitation effects of VR therapy were demonstrated on the distal extremity function of stroke survivors (*n* = 46) in a single‐blinded randomized controlled trial.^162^ Patients were divided into two groups consisting of electronic glove group and conventional intervention group (control) to assess changes in health‐related quality of life indicators. The electronic glove (RAPAEL, Neofect) provided feedback by tracking the posture and the motion of the user's distal limb (Figure 6b). The glove incorporated inertial and bending sensors that measure 3D position of the distal limb and the degree of finger bending. It had the capability to recognize the forearm pronation, ulnar deviation, wrist movement, and finger flexion. The software application then displayed a virtual hand and objects in real time. Fugl–Meyer assessment scores, Purdue pegboard test, Jebsen–Taylor hand function test, and Stroke Impact Scale indicated improvements in the rehabilitation outcomes as compared the control group.

VR reality has been utilized as a pain distraction technology for wound care and physical therapy procedures of burn patients, where pain medications are inadequate.^163^ In a case study, Oculus Rift VR goggles were used to immerse the 3rd degree burn patient into a virtual environment during occupational therapy (Figure 6c). The patient had one occupational therapy session (no VR) on day 1, a VR‐supported occupational therapy session on day 2, followed by a final occupational therapy session with no VR on day 3. Subjective rating of pain intensity results decreased from severely painful to moderately painful when VR was employed. These results were consistent with functional magnetic resonance imaging studies that showed pain‐related brain activity decrease during VR induced pain distraction.^164^

### 2.3 Limitations of Wearable Treatment Approaches

Challenges remain in the development of theranostic systems that enable closed‐loop dynamics and respond correctly to physiological and pathological conditions. The main drawbacks of these technologies for application in point‐of‐care settings arise from the limited integration and miniaturization. Early approaches to treatment of disease by wearables lacked the patient improvement feedback; however, under professional supervision they proved effective, for example, to mitigate Parkinson's disease.^165^ Others have operated under the assumption that the function of the wearable is continuously required, this has proven to be effective for renal failure patients.^166^ Issues with wearables for treatment include low biocompatibility, insufficient conformal contact, inefficient energy consumption, and short lifetime. These factors limit the development of automated systems that can sense physiological stimulus and respond with an accurate therapeutic dosage to create real closed‐loop systems worn long‐term.

### 2.4 Building the Next Generation Wearables

**Figure 7:**
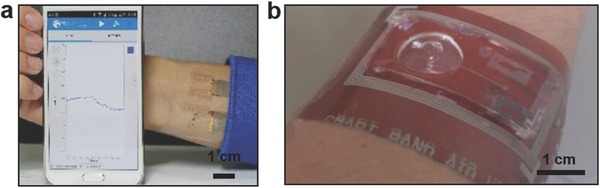
Closed‐loop wearables. a) A wearable device for glucose monitoring and drug‐delivery that can wirelessly communicates with smartphones via Bluetooth. Reproduced with permission.^167^ Copyright 2016, Nature Publishing Group. b) A microfluidic bandage on the arm. The wearable device consists of a capacitive touch detector, a temperature sensor, and a drug delivery pump. Reproduced with permission.^168^ Copyright 2014, Wiley‐VCH Verlag GmbH & Co. KGaA, Weinheim.

Several efforts have been made to solve the limitation of closed‐loop wearables, nevertheless, wearable technologies are still in development. As an example, a promising wearable device featuring a graphene layer doped with gold, and integrated with a serpentine layer of gold mesh for electrochemical monitoring of glucose in sweat was developed (**Figure** **7**a).^167^ The device consisted of a humidity detector, a heater and temperature sensor combined with polyvinyl pyrrolidone microneedles for thermally activated transcutaneous drug delivery. After the patch was laminated on the human skin, the device was activated upon sweat generation in ≈15 min. After reaching 80% relative humidity, the concentration of the glucose and pH was measured in sweat. The microneedles were coated with tridecanoic acid having a transition temperature of 41 °C. As the sensors detected hyperglycemia, the microneedles were thermally activated by melting the tridecanoic acid coating to release *N*,*N*‐dimethylimidodicarbonimidic diamide (Metformin). Experiments on diabetic mice showed suppression of blood glucose concentration by the Metformin‐eluting array as compared to the control group (*P* < 0.05).^167^ However, this system was not suitable for long term delivery of Metformin as the microneedle system consisted of bioresorbable tridecanoic acid coating.

Another challenge is the design of energy efficient theranostic wearable technologies, as the energy requirements tend to be higher as their diagnostic or therapeutic wearable counterparts, nevertheless several examples of energy efficient devices exist.^19^ Recently, a wearable microfluidic bandage incorporating sensors and a drug delivery system was developed (Figure 7b).^168^ This bandage featured sensors, a microfluidic drug delivery pump, a wireless signal transmittance unit, and a microelectoromechanical system on a flexible substrate. The sensing units were fabricated by screen and shadow mask printing techniques in combination with laser cutting. A wireless coil, a capacitive touch sensor, and a temperature sensor were fabricated by patterning silver ink, conductive poly(3,4‐ethylenedioxythiophene)–poly(styrenesulfonate) and a CNT mixture. The drug delivery pump was created by forming PDMS patterns via soft lithography as a semisphere fluid reservoir (0.2 mL). The temperature sensor operated from 20 to 50 °C at a sensitivity of 0.61 °C and functioned at a curvature radius up to 3.6 mm. The threshold pressure to eject a fluid was 3.3 kPa at a rate of 35 nL kPa^−1^, where the bending radius up to 6 mm did not alter the reservoir volume.^168^ The wearable device was used to measure the temperature changes on human arm over time; nevertheless, the drug delivery capability was not tested and the transistors were not integrated within the device, showing limited translation to potential clinical settings.

In creating closed‐loop automated systems for disease monitoring and drug release, the use of sensitive polymers such as chitosan, poly(methacrylicacid), modified poly(acrylamide), sulfonated polystyrenes, and ethylenevinylacetate have enabled the development of systems that interact directly with the tissue microenvironment. These materials can release drugs and modify its physical chemistry based on sensing physiological responses such as temperature, pH, light, electric fields and ultrasound waves.^169^ Nevertheless, challenges remain in accurately controlling the release of the desired drug dosage based on the physiological needs; however studies so far have been limited to characterizing the response of the stimuli‐responsive material. In pathological conditions, the response of materials can be modeled to adjust their release kinetics via the development of several physicochemical formulations. Nevertheless, to develop materials for clinical applications, the development should move from stimuli‐responsive materials to physiologically sensitive materials that act and deliver drug dosages based in organic needs. The ideal material would have to adapt to several circumstances and respond to them in a personalized manner, as most of the current materials present a nonflexible behavior difficult to modulate after its fabrication. This part is fundamental to translate closed‐loop technologies from the laboratory to the clinic as pathological states are fast‐evolving physiological treats that need personalized interventions.

Wearable devices require hardware and software to acquire real‐time data from the user. The data retrieved from sensors are processed, analyzed, displayed, or interpreted to produce actionable information. Hence, hardware and software interactions need to be optimized for each wearable device. An overarching system architecture can be created for wearables, from which particular applications can be derived. As wearables are not user interface centric, plug and play architectures can be conceived when programing or specific data input is required. The application scenarios in disease diagnosis and drug delivery are determining factors for sensor types and processor types in signal analysis and device architecture. Nevertheless, certain components are usually included in all wearable device configurations. The wearables system architecture requirements are driven not only by the continuous data acquisition, but also by the need for a seamless user experience. In healthcare applications, long‐term wear is paramount, also continuous communication to the user for self‐monitoring, and data transfer to a health professional.^170^ As a rule of thumb, system architecture requirements are: (i) long battery life or capability to wirelessly receive power in conjunction with low power consumption, (ii) continuous synchronization within IoT or local networks, (iii) robust sensors with low signal‐to‐noise ratio, (iv) high‐performance data processing, (v) secure or encrypted data transfer, (vii) conformability, comfortability, thin geometry, and (vi) amenable to mass manufacturing at low cost.

#### 2.4.1 Sensor Signal Acquisition, Processing, and Artificial Intelligence Algorithms

A large number of sensors and sensor networks can be integrated into wearable devices. Sensor signal acquisition and processing are key design parameters in wearables. High‐quality raw data is necessary to ensure reliable diagnostic information; the majority of wearables face low‐quality raw data acquisition leading to erroneous health evaluation and diagnosis.^171^ Data processing algorithms have significance in obtaining high‐quality data in multiplexed wearable sensors, where the energy expenditure, heart rate variability, electrodermal response rate, arterial fibrillation, seizure, and stress levels need to be monitored simultaneously (e.g., elderly at high risk of heart disease). The first on‐chip data processing necessary for reliable data collection is the identification and reduction of motion artifacts, for which various algorithms have been developed. For example, using data from a 3D accelerometer, such artifacts can be reduced in ECG measurement systems or pulse oximetry.^172^ These adaptive filter algorithms typically consist of least mean squares algorithms and variations of the same such as adaptive step‐size, or recursive LMS.^173^ The next layer algorithms correspond to feature extraction for the determination of sensory parameters. Since some biological signals oscillate, it is common to retrieve time frequency domain algorithms or fast Fourier transforms.^174^ Common feature extraction and classification in wearable devices include dimension reduction such as principal component analysis, Laplacian eigenmaps, and independent component analysis.^175^ Hilbert and wavelet transforms can enhance noise reduction and extract time‐independent features.^176^ Multiscale analysis algorithms have also been utilized for long‐term feature collection and analysis.^177^ Other techniques include forward–backward sequential search using multilayer preceptors and nearest neighbor classifiers, which are machine learning algorithms.^178^ Full artificial intelligence algorithms are expected to be applied for integral signal processing in medical wearable devices.

Key trends for the development of wearable devices are multiplexed sensors, continuous connectivity, low power consumption, and novel material integrations. However, multiplexed sensing is challenging due to different material‐body interfaces and probe positioning. Multiple interconnected sensors can be positioned on body areas of importance. Short‐range radio signals for sensor networks have been proposed to reduce power consumption and achieve continuous sensor communication.^179^ Preinstalled algorithms in microcontrollers can meet the requirements of multiplexed sensor systems. For example, sensor system‐on‐chip (Imec) devices are capable of measuring ECG, respiration by bioimpedance, 3D accelerometer data, all with preloaded signal analysis algorithms.^180^, ^181^ Other examples of system‐on‐chips include monitoring of cardiac rhythm,^182^ energy expenditure,^180^ and the onset of seizures.^183^ Such architectures have evolved to operate at low voltages, low power, and fully integrate into standard chip architectures.

#### 2.4.2 Energy Storage and Charging

**Figure 8:**
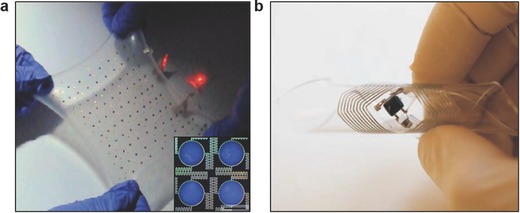
Flexible batteries and wires energy transfer for wearable devices. a) Photograph of a biaxially stretched (300%) battery with serpentine interconnects. The inset shows the electrode pads and interconnects of the battery. Scale bar = 2.0 mm. Reproduced with permission.^187^ Copyright 2013, Nature Publishing Group. b) A flexible liquid alloy coil for wireless power transfer. Reproduced with permission.^194^ Copyright 2015, Nature Publishing Group.

Flexible battery constructs and energy management systems are key design components in wearables.^184^ High‐energy density batteries designed for wearable devices should withstand bending, folding, and stretching while being amenable to miniaturization and easily integrated (e.g., weaving).^185^ The flexibility of wearable devices mainly depends on the mechanical properties of the electrodes.^186^ A rechargeable Li‐ion battery has been fabricated from low‐modulus silicone elastomers as substrates, segmented active components, and interconnected structures.^187^ The battery consisted of pouch cells of 100 photolithographically patterned Al and Cu disks connected in parallel with molded pads of cathode and anode consisting of LiCoO_2_ and Li_4_Ti_5_O_12_. A spacer was created in the system, where a gel electrolyte injected into this gap acted as a medium for ionic transport. This system was encapsulated in a polyamide layer and packaged in acryloxy perfluoropolyether elastomer. The battery had a stretchability of 300% with a capacity density of ≈1.1 mAh cm^−2^ (**Figure** **8**a). A wireless charging system was designed with 1 mm spaced coils, where the received power by the receiving coil was 9.2 mW, showing a DC output of 3.0 V with an efficiency of 4.9%.^187^ Recently, fiber‐shaped Li‐ion batteries were fabricated from yarns made of multiwalled carbon nanotube/lithium oxide composite,^188^ and Si‐carbon nanotubes.^189^ Furthermore, lithium polymer technology is the current benchmark for commercial products; however, Zn–air and Li–air batteries may offer longer energy storage.^190^ Zn–air batteries require the diffusion of atmospheric oxygen into a porous carbon electrode and consist of zinc metal as an anode and air electrode as cathode, which is separated into catalytic activity layer, gas diffusion layer, and a separator.^191^ Rechargeable Li–air batteries are also promising candidates as their theoretical and practical specific energies can reach up to 12 000 and 4000 Wh kg^−1^.^192^ As these batteries use Li as anode and oxygen is sourced from air to function as a cathode, they requires air dehydration membranes.^193^

To achieve wireless power transfer in wearable devices, stretchable microfluidic devices have been fabricated.^194^ Liquids in microfluidic channels can deform without significant hysteresis and flow without discontinuity upon bending (Figure 8b). The maximum power efficiency was ≈10% corresponding to receiving power of 0.47 W from a transmitting power of 4.6 W. The resulting wireless energy transfer device has the capability to function after 1000 cycles of strain (25%).^194^ In another study, conductive yarns of silver‐coated copper wire and polyester filaments have also been modified to transfer wireless energy.^195^ The silver‐plated copper wire (40 µm) was wrapped around polyester yarn of 75 denier with 150 twists m^−1^ and the copper filament was then turned 700 twists m^−1^. To construct the final yarn, three strands were piled together to create a yarn density of 547 denier with a diameter of 0.3 mm resulting in a resistance of 89 mΩ cm^−1^. The number of turns (50) to construct the coil was designed to have a resonant frequency of 6.78 or 13.56 MHz for operation in medical (ISM) band.^195^ An average power transmission efficiency of 45% was achieved. Highest transmission was achieved at 6.5 cm with no interference by the skin contact with the yarn.

#### 2.4.3 Displays

**Figure 9:**
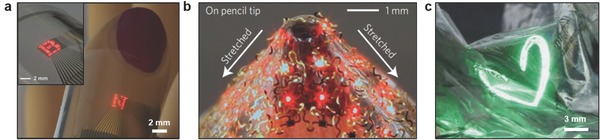
Flexible displays for wearable devices. a) Photograph of flexible display having a 16 × 16 array of ILEDs on a sheet of plastic (PET) wrapped around a thumb. The inset shows the display around a cylindrical glass tube (radius ≈12 mm). Reproduced with permission.^194^, ^197^ Copyright 2009, American Association for the Advancement of Science. b) An image of an array of ILEDs array (6 × 6), stretched on the sharp tip of a pencil. Reproduced with permission.^198^ Copyright 2010, Nature Publishing Group. c) A photograph of green QLEDs laminated on wrinkled Al foil. Reproduced with permission.^199^ Copyright 2015, Nature Publishing Group.

The physical and geometrical optoelectronic design constrains in semiconductor wafers can be reduced by creating inorganic light emitting diode, display, and photodetector layouts on flexible polymeric substrates.^196^ One approach is to use sacrificial substrate to construct electronic circuitry. For example, epitaxial semiconductor layers were produced on GaAs wafers, vertically etched, and these layers were separated from the wafer by etching the AlAs layer.^197^ These produced layers were then transfer printed on a temporary substrate coated with a trilayer of epoxy‐polyimide‐poly(methylmethacrylate) (PMMA). After interconnections, structural bridges, and encapsulating layers were formed, the PMMA layer was dissolved and the system was transfer printed over an elastomeric sheet such as PDMS substrate. µILEDs were connected by serpentine‐shaped ribbons that served as electrical interconnects and structural bridges (**Figure** **9**a). The applied strains to these devices before and after stretching in horizontal and diagonal directions could reach 48% and 46%, respectively. The device characteristics were stable after 100 000 cycles in the horizontal direction, and the area expansion could reach 85% without any structural failures (Figure 9b).^198^ Additionally, wearable devices should preferably incorporate deformable full‐color displays for conformal integration on curved surfaces. Recently, a quantum dot based LED array was fabricated by intaglio transfer printing onto flexible and curved surfaces (Figure 9c).^199^ This high‐resolution printing process involved creating intaglio trench and nanocrystal layers that were transfer printed on different substrates. Quantum dot layers were first deposited on a donor substrate and then lifted with a PDMS transfer stamp and contacted (<50 g cm^−2^) on the intaglio trench. The resulting red–green–blue LEDs had a resolution of 2460 pixels in^−1^. These LEDs showed an electroluminescence performance of 14 000 cd m^−2^ with 7 V, and operated over 1000 deformation tests.^199^ Recently, origami‐inspired bazel‐lens transistor arrays have been fabricated to create stretchable and foldable electronics.^200^ The substrates composed of metal films and nanowires connected with elastomeric PDMS joints. Wearable devices, however, are not screen‐centric and alternatives are being proposed for user interfaces that rely on connectivity rather than immediate display of information.

#### 2.4.4 Comfort and Conformity

High level integration on the chip ensures compact organization of all components for medical wearables. Advanced packaging of electronics requires novel device assembly and chip building methodologies such as stacked dies (3D integrated circuits), multichip packages, or full systems in package.^201^ Second, wearables require a small footprint to seamlessly integrate with the user's body or clothing as functionality affects the wearing comfort.^202^ Finally, innovative materials are required, not only to enhance conformity but to ensure body‐device interaction and achieve high‐quality signal acquisition. Analog sensor‐device interfaces are key for optimal health assessment and the quality of signals being transmitted. As wearable devices evolve, new materials will be required for individual type of sensors (impedance, conductive, optical), reduce motion artifacts, contact with dry electrodes, identification of physiological differences, and measurement at nonoptimal positions. For example, polymers have been utilized for biointegration and obtaining optimal contact in wearable devices.^203^ Strain‐gauge sensors featuring high‐aspect‐ratio Pt‐coated polyurethane nanofibers have been developed to mimic the mechanical properties of the human skin.^204^ Piezoelectric crystals, optical components, and hybrid interconnects have been proposed for microelectronic architectures.^205^ Additionally, electronic components can bend and adapt to the wearer's activity leading to fiber‐ and textile‐based wearable electronics and sensor components.^4^, ^206^ User experience with wearables has been poor due to the ad hoc design of user interfaces, requiring manual control.

## 3 Wearables Market

**Figure 10:**
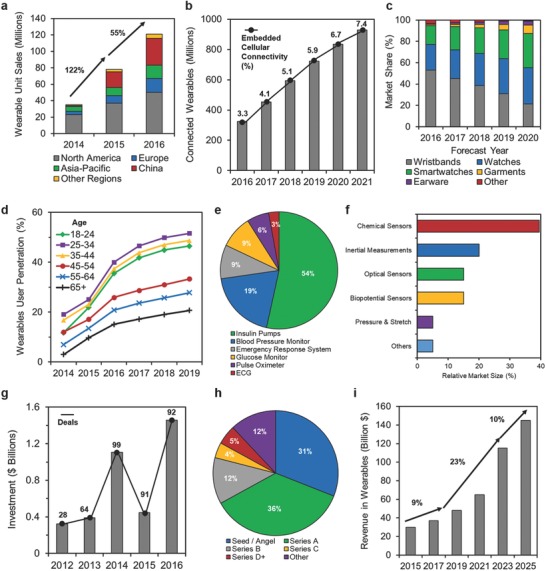
The wearables market. a) Unit sales worldwide by region. b) Number of connected wearable devices worldwide. c) Wearables forecast by product type worldwide. d) User penetration by age group. e) Regulated wearable devices by product category in the United States in 2016. f) Sensor type market projection of wearables market by 2020. g) Annual investment and the number of deals. h) Investment deal breakdown by stage (2013–2014). i) Revenues in wearables worldwide.

With the emergence of “quantified‐self” movement, technology companies have motivated their customers to seek wearable products in social pull marketing strategies. Consumer‐driven demand for Apple Watch, Samsung Gear, and Fitbit Wristband in the wearables market has prompted mass public attention.^207^ These wearable technologies aim to improve physical performance and form positive exercise and diet habits through digital persuasion. Such wearable devices may offer social influencing or gamification of the exercise through creating challenges as well as virtual rewards to improve physical performance.^208^ Worldwide market of wearable device sales has reached 125 million units in 2016, indicating a 55% increase as compared to 2015; and the sales are estimated to reach 237 million devices by 2020 (**Figure** **10**a).^209^ North America had the highest regional share of sales (41%) in 2016, followed by China (27%) and Europe (14%). In 2016, 325 million connected devices were in use worldwide and the users are projected to reach 929 million by 2021 with a Compound Annual Growth Rate (CAGR) of 23% (Figure 10b).^210^ Embedded cellular connectivity in wearables will subtly increase from 3.3% in 2016 to 7.4% in 2021. Wristbands had the largest market segment of unregulated wearables (53%) in 2016, followed by basic watches market (23%), which is shifting to smartwatches market (17%) (Figure 10c).^211^ The main market segment focus of these consumer products is within connected fitness and sports monitoring technologies for tracking of exercise performance metrics.^212^ The customer base of wearable devices is mainly below the age of 44, and the penetration into older age groups has been limited (Figure 10d). Today one in five individuals in the United States own a wearable device that are mainly activity trackers.^213^ However, wristbands market is projected to decrease due to the customer base transition to smartwatches that will offer new sensors and wider applications.^211^ Additionally, smartwatches will see an increase in market penetration due to the advances in cellular connectivity. 5G communication will be a crucial part of these wearable technology paradigm shift, in which customers will demand faster and secure data connection through their wearable devices, smartphones, and telehealth.^214^ Smart garments market is expected to grow due to the integration of electronics and traditional clothing industry to create textiles and connected apparels for application in fitness, drug delivery, and clinical trials.^5^, ^215^

In the regulated wearables market, insulin pumps dominate the market with a share of 54% in the United States in 2016 (0.7–1.0 million pump users worldwide) (Figure 10e).^216^ Blood pressure monitoring devices have the second largest regulated wearables market segment (19%), which can be attributed to the hypertensive patients, which correspond to 16–37% of population globally.^217^ Furthermore, chemical sensors are projected to have 40% of the market share followed by the inertial measurement sensors (20%) by 2020 (Figure 10f). This trend will be driven by the advances in the development of low‐power microelectromechanical systems and biochemical continuous monitoring systems that do not require frequent calibrations.^218^

Over $3.7B has been invested in emerging startups in wearables since 2012 corresponding to an increase of 351% (Figure 10g).^219^ The majority of investments were angel/seed (36%) and series A (36%) funding and more than two thirds of the deals were in this space largely driven by True Ventures, Andreesen Horowitz, Khosla Ventures, First Round Capital, Bessemer Venture Partners, and Intel Capital (Figure 10h).^220^ Largest deals in 2016 included Magic Leap ($793M, series C), Jawbone ($165M, series F), Thalmic Labs ($120M, series B), Razer ($75M, series C), and Chrono Therapeutics ($48M, series C). California has been dominant in the wearables space, representing 77% of the total funding and half of the companies were funded in this region. The sales have been mainly in North America and Europe as well as in China and the market is projected to grow in these regions. The wearables industry involving the device sales, software, and data services has generated $33B in 2016. A limited 10% growth is expected until 2018 when the market would reach $40bn, followed by an accelerated growth of 23% through to over $100B by 2023 and, a slower growth (11%) to reach over $150B by 2025 (Figure 10i).^221^

## 4 Regulatory Pathway of Wearables

Wearable devices are regulated in the same way as any other medical device based on medical use intent. These regulations are in place to ensure that wearables do not harm patients during device use or result in the long‐term effects. Wearable devices are associated with numerous risks to patients and their use in conjunction of regular software updates introduces an additional layer of risk factor and complexity. FDA has issued a guideline, entitled “Mobile Medical Applications Guidance for Industry and FDA Staff” in 2015 to clarify its stance and evaluate the intended use and functionality of wearable devices.^222^ For example, if a wearable device monitors blood pressure of a patient with heart disease, it would fall under these regulations. However, if a patient uses the wearable device to record his/her calories during his exercise, it will not be regulated. As another example, wearable devices providing electrocardiograph measurements must acquire physiological data and have a comparable performance with commercial electrocardiography devices. FDA regulations do not distinguish between platforms or device size, but focuses on the medical function and intended use.^223^ As the quantified‐self movement rises rapidly, there is a genuine concern among regulatory bodies for the unintended use of wearables developed for exercise and entertainment purposes. For example, a patient may use an inaccurate exercise‐focused calorie‐counting wearable device to manage his/her diet that can lead to unintended health consequences. Currently, most wearable devices in the market lack controlled experiments. The majority of the manufacturers have not published empirical data and accuracy values, as this may originate from large error values. Another significant issue is related to automated software updates, which may introduce bugs to the wearable devices. There is not only lack of evidence on the performance of the claims by some device manufacturers, but most of them rely on the approvals granted for the individual sensors. Controlled studies are required for performance evaluation under clinical and point‐of‐care settings. Furthermore, the effects of electromagnetic waves on human health have been widely studied in smartphones. However, the electromagnetic effects of wearable devices having closer contact to the body in a continuous manner have not been studied. Electromagnetic radiation may affect transitions between energy states of molecules including DNA and proteins, as well as disruption of the neuron firing.^223^ Hence, the exposure of the continuous electromagnetic fields on tissues and biological mechanisms warrant further research.

## 5 Connecting Wearables to Clinics

The majority of commercial wearables are used by individuals who are healthy and seek methods to quantify their fitness progress.^224^ However, potential medical applications of wearables are broader ranging from point‐of‐care diagnostics to treatment. Wearables have applications in home monitoring to evaluate disease progression, patient's response to medication, or health recovery after surgical intervention. In the case of chronic diseases, wearables have the advantage of logging patient data in real time. For example, the severity of depression can be measured by quantifying physical activity, conversation timelines, and sleep duration.^225^ Furthermore, wearables can allow early diagnosis of sleep apnea, which can be treated by improving sleep quality.^226^ The analysis of the body movements may allow diagnosing Parkinson's disease at an early stage.^227^ Wearables also have applications in the home monitoring of a wide range of long‐term medical conditions via electronic self‐reports, web‐based counseling, and e‐mail feedbacks to facilitate positive behavior. These medical conditions may include post‐traumatic stress disorder,^228^ anxiety,^229^ panic disorders,^230^ obesity,^231^ and asthma.^232^ The key advantage of wearables is to provide instant feedback for each patient, replacing subjective questionnaires to obtain clinical data.

Real‐time monitoring of vital signs may reduce the burden on hospital appointments, and FDA‐cleared devices in the market are already used for this purpose. An actigraphy measurement device, having a triaxial accelerometer and a light intensity sensor to monitor luminous intensity has been used to monitor limb or body movements to assist specialists in diagnosing sleep disorders.^233^ It was successfully tested in measuring sleep quality and physical activity to assess dementia progression in older adults.^234^ Another wrist‐worn FDA‐approved product is an electronic diary for collecting patient reported outcome. Obtaining subjective data is useful for ecological momentary assessments,^235^ but also to monitor appetite,^236^ satiety,^237^ pain intensity,^238^ analgesic use,^238^ sleep onset and latency,^239^ and physical activity.^240^ Additionally, another FDA‐cleared, wearable multiparameter vital sign monitoring device records and transmits electrocardiography (ECG), heart rate, respiratory inductance plethysmography, calorific expenditure, posture/activity, skin/core temperature, and oxygen consumption.^241^ Experimental studies to monitor physiological signals and ECG for detecting arrhythmias have been successfully carried out using this type of technologies.^242^ As compared to the standard laboratory equipment, the heart and respiratory rate values were clinically acceptable (95% limit of agreement) for application in ambulatory monitoring. There are ongoing clinical trials, for example, to monitor patients with amyotrophic lateral sclerosis,^243^ and new wearables are entering commercialization such as electronic stamps (tattoos) to monitor temperature, heartrate, and glucose.^244^ Hence, wearables are creating new market segments for the real‐time analysis of patient's health status. Software components are integral part of most wearable technologies, regulations, and compliance of such products is paramount for the integration of wearables into the healthcare systems.^245^

## 6 Patient‐Centered Wearables

A limited number of randomized and controlled studies has been carried out to measure the efficacy of wearable devices on consumer behavior and health. Evaluations of different wearable devices (e.g., fitness trackers) for monitoring physical activity showed inaccurate variations with error margins up to 25%.^246^ These error margins are comparable to those of mobile medical applications having failure rates up to 30%.^247^ In a recent study, the ECG data of Fitbit fitness trackers (Surge and Charge HR) equipped with PurePulse technology were tested among 43 healthy adults.^248^ Fitbit fitness trackers were off by ≈20 beats per minute during medium/high‐intensity exercise. Another study that evaluated the step counts recorded by Fitbit One indicated valid results at multiple running speeds on a treadmill; however, the measurement of distance travelled was inaccurate.^249^ Similarly, commercial fitness trackers tend to overestimate the calories burnt by 30%, which puts the patients at risk who adjust their diets and exercise plans to maintain or lose weight.^250^ Over‐reliance on wearable devices in particular with patients with chronic, life‐threatening conditions (e.g., diabetes) may result in false sense of security and misdiagnosis.^251^ Additionally, the measurement of metabolic signals and their delivery in real‐time to the patient does not necessarily improve the outcomes. A randomized trial of self‐tracking of blood glucose concentration in noninsulin treated type‐2 diabetics showed no convincing improvement.^252^ Another study in newly diagnosed type‐2 diabetic patients indicated that self‐monitoring of glucose was associated with depression.^253^

While pedometers and smartphone applications have been shown to decrease blood pressure and body mass index, these interventions were not proven to achieve continued behavioral changes during the intervention.^254^ Additionally, the personality of the patient influences the efficacy and perceived performance of the wearable device.^255^ The short‐term behavior change in the exercise activity of the patients can be attributed to the novelty effects.^256^ One third of wearable users completely discontinue using their devices after six months.^257^ Moreover, the quality of life indicators including cost‐effectiveness, mental and social functioning are rarely reported in mobile health monitoring studies, where the existing study criteria and methodologies need significant improvement.^258^ While the advances in the development of wearable devices will undoubtedly grow in the upcoming decades, randomized clinical studies are needed to evaluate their real impact in patient care. In the near future, wearable devices not only will be preventive, diagnostic, and therapeutic technologies, but also will enable continuous acquisition of data to monitor disease progression, drug response and assess clinical trial efficacy.

## Conflict of Interest

The authors declare no conflict of interest.
